# Antifungal Activities of Peptides Derived from Domain 5 of High-Molecular-Weight Kininogen

**DOI:** 10.1155/2011/761037

**Published:** 2011-09-14

**Authors:** Andreas Sonesson, Emma Andersson Nordahl, Martin Malmsten, Artur Schmidtchen

**Affiliations:** ^1^Division of Dermatology and Venereology, Department of Clinical Sciences, Lund University, 221 84 Lund, Sweden; ^2^Department of Pharmacy, Uppsala University, 751 23 Uppsala, Sweden

## Abstract

In both immunocompromised and immunocompetent patients, *Candida* and *Malassezia* are causing or triggering clinical manifestations such as cutaneous infections and atopic eczema. The innate immune system provides rapid responses to microbial invaders, without requiring prior stimulation, through a sophisticated system of antimicrobial peptides (AMPs). High molecular weight kininogen (HMWK) and components of the contact system have previously been reported to bind to *Candida* and other pathogens, leading to activation of the contact system. A cutaneous *Candida* infection is characterized by an accumulation of neutrophils, leading to an inflammatory response and release of enzymatically active substances. In the present study we demonstrate that antifungal peptide fragments are generated through proteolytic degradation of HMWK. The recombinant domain 5 (rD5) of HMWK, D5-derived peptides, as well as hydrophobically modified D5-derived peptides efficiently killed *Candida* and *Malassezia*. Furthermore, the antifungal activity of modified peptides was studied at physiological conditions. Binding of a D5-derived peptide, HKH20 (His^479^-His^498^), to the fungal cell membrane was visualized by fluorescence microscopy. Our data disclose a novel antifungal activity of D5-derived peptides and also show that proteolytic cleavage of HMWK results in fragments exerting antifungal activity. Of therapeutic interest is that structurally modified peptides show an enhanced antifungal activity.

## 1. Introduction

The innate immune system provides rapid responses to microbial invaders without requiring prior stimulation. It is a sophisticated system comprising epithelial barriers, white blood cells, various peptides and proteins including antimicrobial peptides (AMPs), as well as lipids and other chemicals, such as reactive oxygen species [1–4]. At present, approximately 1600 AMPs of different origin have been identified (http://aps.unmc.edu/AP/main.php). A blend of endogenous AMPs, including the cathelicidin LL-37/hCAP18, *β*-defensins, RNase 7, various dermcidins, as well as psoriasin, are found in skin [5–9]. Peptides of the histatin family, like many other histidine-rich AMPs [10], are known to harbor antifungal activities. Histatin 5 is secreted by the salivary glands and exerts its activity by entering the fungal cytoplasm targeting the mitochondria [11, 12]. Bacterial, fungal, and mammalian cell surfaces have structural differences which may explain a certain degree of selectivity for AMP action. Specifically, bacterial surfaces contain many anionic components, including LPS and anionic lipids of Gram-negative bacteria, as well as teichoic and teichuronic acids of Gram-positive bacteria. Similarly, glucan, chitin, mannoprotein, and a blend of other cell wall proteins and polysaccharides contribute to a negative surface potential of fungal surfaces. Beyond the outer cell surface, AMPs interact with the plasma membrane. Furthermore, fungal membranes contain mostly zwitterionic lipids (e.g., phosphatidylcholine), but also ergosterol, whereas bacterial membranes comprise various acidic phospholipids (phosphatidylglycerol, phosphatidylserine, and cardiolipin), which confer a negative charge facilitating AMP binding and sometimes also defect formation [13–17]. AMPs may preferentially target fungi, bacteria, or mammalian cells, or combinations thereof, and the interaction and disruption of the target membrane is dependent on a number of parameters, including hydrophobic and ionic interactions, as well as influence of oligomerisation, the ionic environment, and the presence of plasma proteins [13–17]. Many endogenous AMPs are not only antimicrobial, however, but have many additional functions, including antiendotoxin or antitumor effects, anti-inflammatory properties, mediation of chemotaxis, or activation of the immune system [7]. Although AMPs display a range of conformations, such as *α*-helices and *β*-sheets, they all share common properties such as amphipathicity, cationicity, and the propensity to bind to negatively charged molecules such as negatively charged phospholipids, glycosaminoglycans, and heparin [18–21]. Concerning bacterial killing, the mechanisms of AMP action are complex and are often divided into membrane active and nonmembrane disruptive mechanisms [15, 22, 23]. 

*Candida albicans* is normally colonizing human skin as well as other epithelial surfaces. However, in both immunocompromised and immunocompetent patients, *Candida *may cause or trigger several clinical manifestations such as cutaneous infections, atopic eczema, oroesophageal candidiasis, candida vaginitis, urinary candidiasis, and disseminated candidiasis and candidemia [24–26]. *C. albicans* is the most prevalent yeast in clinical infections, but non-*C. albicans* species are increasingly causing severe infections such as candidemia, especially in patients suffering from endogenous or drug-induced immunosuppression and critically ill patients [27]. The yeast *Malassezia* is a member of the cutaneous microbial flora, and involved in several dermatologic diseases such as atopic dermatitis, pityriasis versicolor, and seborrheic dermatitis [28], and also reported to cause invasive infections in premature infants [29]. Importantly, some *Candida* isolates are known to have reduced susceptibility to antifungal drugs [24], which makes other strategies based on antifungal proteins and peptides interesting alternatives in the treatment of fungal infections. 

High molecular weight kininogen (HMWK) and components of the contact system have previously been reported to have the capacity of binding to the surface of a blend of human pathogens, including *Candida*, leading to activation of the contact system [30–32]. The 120 kDa glycoprotein HMWK contains six domains with different functions. Domain 3 and 5 have been previously reported to harbor epitopes, that may function as antimicrobial peptides on release [33, 34]. Furthermore, activation and degradation of HMWK leads to kallikrein-mediated generation of bradykinin [35]. However, endogenous generation of active kinins can also be mediated by the cooperative action of mast-cell tryptase and neutrophil elastase. Kinins can also be proteolytically generated from plasma HMWK by the action of microbial and dust-mite proteases [36, 37].

 In the present study, we have investigated whether rD5, D5-derived peptides, and structurally modified D5-derived peptides exert antifungal activity. In addition, the capability of these modified peptides to work as antifungal peptides at physiological conditions was elucidated. Our data disclose a novel antifungal activity of D5-derived peptides and also demonstrate that HMWK, when subjected to degradation by neutrophilic enzymes, may release fragments with antifungal activity.

## 2. Materials and Methods

### 2.1. Proteolytic Cleavage of HMWK and Detection of Fragments

To investigate the neutrophil elastase effects on HMWK, the protein (32 *μ*g) was incubated at 37°C for 30 and 60 minutes with human leukocyte (neutrophil) elastase (HLE) (24 mU) or freeze/thaw-disrupted polymorphonuclear neutrophils (PMNs) (18 *μ*L, 1×10^7^  cells/mL) in a total volume of 40 *μ*L. HMWK (32 *μ*g) incubated at 37°C for 60 minutes was used as a control. Ten *μ*L of the material was analyzed by sodium dodecyl sulphate-polyacrylamide gel electrophoresis (SDS-PAGE) on a 16.5% Tris-Tricine gel (Bio-Rad Laboratories, Hercules, Calif, USA) under reducing conditions. 

**Figure 1:**
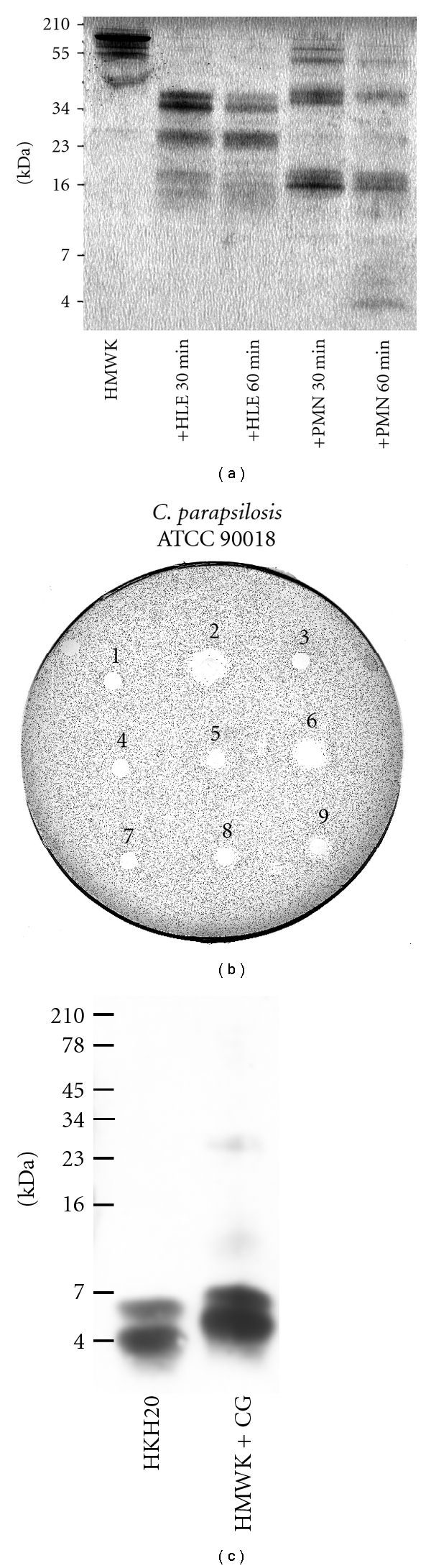
Degradation of HMWK generates antifungal peptides. (a) Intact HMWK and cleavage products from different incubations (indicated below, human leukocyte elastase, HLE, and polymorphonuclear neutrophils, PMN), were analyzed by SDS-PAGE (16.5% Tris-Tricine gel). (b) Antifungal activity of HMWK cleavage products is shown as clear zones corresponding to inhibition of fungal growth in RDA. Cleavage of HMWK was performed for 30 and 60 minutes at 37°C. (1) control, buffer (10 mM Tris, pH 7.4). (2) reference, LL-37 (6 *μ*L at 100 *μ*M). (3) control, HMWK. (4) control, human leukocyte elastase (HLE). (5) and (6) HMWK incubated with HLE for 30 and 60 minutes, respectively. (7) control, polymorphonuclear neutrophils (PMN). (8) and (9) HMWK incubated with PMN for 30 and 60 minutes, respectively. Data represents the results from a single representative experiment out of three. (c) HMWK was subjected to Cathepsin G, producing cleavage products recognized by polyclonal antibodies against HKH20 by Western blot analysis. In the panels, molecular mass markers are indicated on the left.

For detection of fragments generated by Cathepsin G, HMWK (16 *μ*g) was incubated with Cathepsin G (9 *μ*U) at 37°C for 30 minutes and analyzed by Western blot (Figure 1(c)). The proteins were further transferred onto nitrocellulose membranes (Hybond-C, Amersham Biosciences, UK). The membrane was blocked using 3% (w/v) skimmed milk (ICN Biomedicals Inc., Ohio, USA), washed, and then incubated for 1 h with rabbit polyclonal antibodies (Innovagen AB, Lund, Sweden) against the peptide HKH20 (at 1 : 1500), washed again and incubated with horseradish peroxidase-conjugated swine antirabbit secondary antibodies (DAKO A/S, Glostrup, Denmark) (1 : 1000). The proteins reacting with the antibodies were detected by using enhanced chemiluminescence ECL Western Blotting Detection Reagents Kit (Amersham Biosciences, Buckinghamshire, UK).

### 2.2. Peptides and Biological Materials

The peptides (Table 1), LL-37, histatin 5, and the synthetic peptides HKH20, GKH17, GGH20, GHG20, GHG21, KHN 20, GKH17WWW, GKH17WWWWW, and Texas Red-conjugated HKH20, were all synthesized by Innovagen AB (Lund, Sweden). The purity (>95%) and molecular weight of these peptides were confirmed by mass spectral analysis (MALDI-TOF Voyager, Applied Biosystems, Foster City, Calif, USA). Citrate plasma (fresh frozen) from healthy volunteers was obtained from the blood bank at Skåne University Hospital, Lund, Sweden. Neutrophils were prepared by routine procedures (Polymorphprep*™*, AXIS-SHIELD PoC AS, Oslo, Norway) from blood of healthy human donors. The cells were disrupted by freezethawing followed by the addition of 0.3% Tween 20. Human leukocyte elastase (4 mU *μ*L^−1^) was obtained from Calbiochem (San Diego, Calif). The purification of recombinant domain 5 was done as previously described [33]. Briefly, the expression vector (pET25b) (Novagen, Inc., Madison, Wis) containing rD5 was expressed in *Escherichia coli* strain BL21 (DE3), generously provided by Herwald et al. [38]. *E. coli* was subjected to induction of protein production by 1 mM isopropyl thio-*β*-D-galactoside, and, after 3 hour incubation at 30°C, the bacteria were harvested by centrifugation. The pellet was resuspended in sonication buffer (50 mM phosphate, 300 mM NaCl, pH 8.0), and bacterial cells were lysed by repeated cycles of freeze thawing. The supernatant of the lysate was mixed with 2 mL of nickel-nitrilotriacetic acid-Sepharose (Qiagen GmbH, Hilden, Germany), equilibrated with sonication buffer, and incubated on rotation for 1 h at room temperature. The Sepharose gel was loaded into a column washed, and the rD5 protein was eluted as previously described [33].

### 2.3. Determination of Antifungal Activity

**Figure 2:**
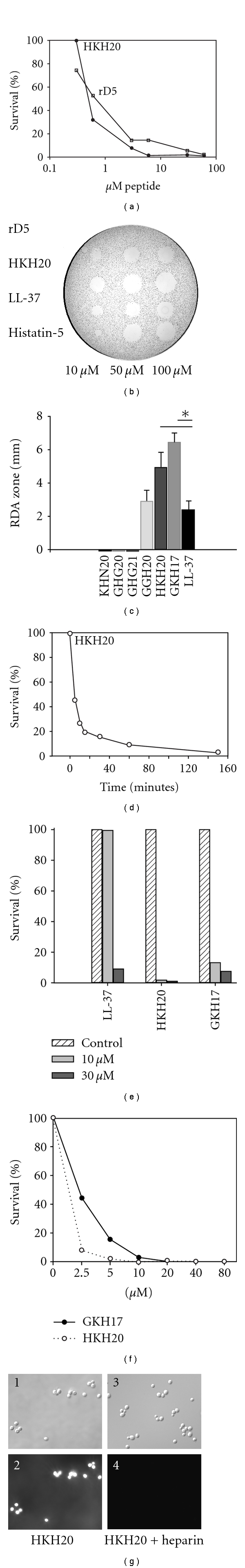
Antifungal activity of rD5 and the D5-derived peptide HKH20. (a) In viable count assay, antifungal activities of rD5 and HKH20 were detected. 1 ×10^6^ cfu mL^−1^  of *C. parapsilosis* ATCC 90018 was incubated in 50 *μ*L with rD5 or HKH20 at concentrations ranging from 0.3 *μ*M to 60 *μ*M. (b) *C. parapsilosis* ATCC 90018 was subjected to rD5, HKH20, and the reference peptides LL-37 and Histatin-5 in RDA, each well was loaded with 6 *μ*l of peptide at the indicated concentrations. (c) The indicated D5-derived peptides (6 *μ*L each at 100 *μ*M) were tested in RDA in low salt conditions against *C. albicans* ATCC 90028. GGH20, HKH20, and GKH17 showed antifungal activity. For comparison, the reference peptide LL-37 is shown. * indicates a statistically significant difference (*P* < 0.05) in comparison to LL-37. (d) Time-dependent killing of *C. parapsilosis* ATCC 90018 by 30 *μ*M HKH20 was analyzed by viable count assay. (e) *M. furfur* ATCC 44342 in a suspension of 1-2 × 10^5^ cells/mL was added to buffer only (control, striped) or subjected to 10 *μ*M (light gray) or 30 *μ*M (dark gray) of the AMPs LL-37, HKH20, or GKH17 and incubated for 24 h. (f) *M. furfur* ATCC 44342 was subjected to increasing concentrations of GKH17 (filled circle) and HKH20 (open circle) and incubated for 24 h, and the percentage of survived yeast cells is shown. (g) Fluorescence microscopy shows binding of Texas Red-labeled HKH20 peptide (10 *μ*M mL^−1^) to *Candida* cells. Panel 4 shows yeast cells incubated with heparin and Texas Red-labeled HKH20. Images in panel 2 and 4 were recorded using identical instrument settings. The corresponding Nomarski images are shown in panels 1 and 3. Data represents the mean of at least duplicate determinations from a single experiment representative of at least two experiments.

**Figure 3:**
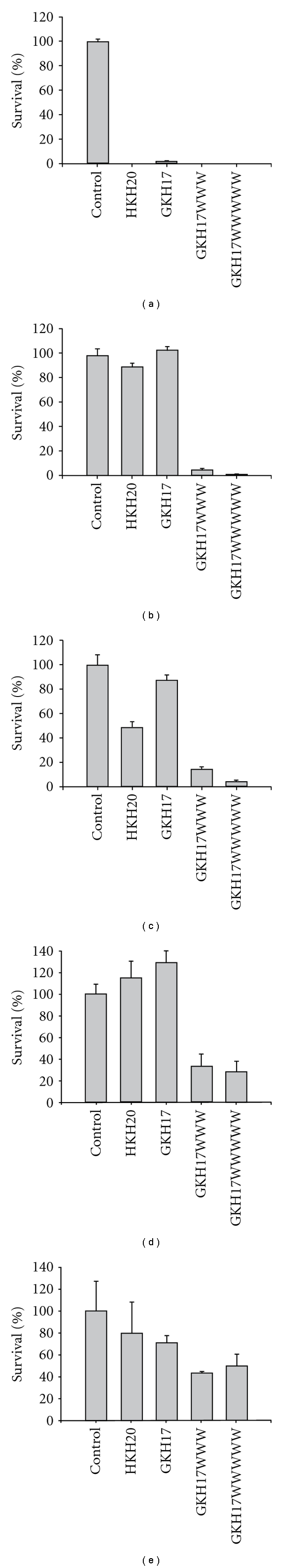
Antifungal effects of the D5-derived and the modified peptides. (a)–(e) The D5-derived peptides HKH20, GKH17, and modified variants of GKH17 were tested against *C. parapsilosis* ATCC 90018 in viable count assays at different physiological conditions. *Candida* cells were subjected to 30 *μ*M of the peptides at (a) low salt conditions (10 mM Tris, pH 7.4), (b) physiological salt conditions (0.15 M NaCl in 10 mM Tris, pH 7.4), (c) in presence of 20% human plasma (0.15 M NaCl, 10 mM Tris, pH 7.4), (d) in presence of 50% human plasma (0.15 M NaCl, 10 mM Tris, pH 7.4), and (e) in presence of 50% human serum (0.15 M NaCl, 10 mM Tris, pH 7.4). Most of the antifungal activities of the D5-derived peptides HKH20 and GKH17 were lost, whereas the activities of the modified variants of GKH17 peptides were retained in physiological salt and presence of human plasma or serum. Data represents the mean ± (SD) of triplicate determinations from a single experiment representative of at least two experiments.

*C. albicans *ATCC 90028 and *C. parapsilosis* ATCC 90018 were obtained from the Department of Bacteriology Lund at Skåne University Hospital. Cells were precultured overnight on Sabouraud-dextrose-agar (Difco, Detroit, Mich) at 28°C, inoculated in 10 mL yeast extract-peptone-dextrose (YPD) broth (Sigma-Aldrich, St. Louis, Mo USA) at 28°C, and grown to mid-logarithmic phase. The cells were washed three times and diluted in 10 mM Tris, pH 7.4. Fifty *μ*L of a suspension containing 1 × 10^6^ fungal colony-forming units (cfu)/mL was incubated at 28°C for two hours with rD5, HKH20 at concentrations ranging from 0.3 *μ*M to 60 *μ*M, or the peptides HKH20, GKH17, GKH17WWW, and GKH17WWWWW at a concentration of 30 *μ*M. To quantify the fungicidal activity, serial dilutions of the incubation mixture were plated onto Sabouraud-dextrose-agar plates (Difco, Detroit, Mich) and incubated for 48 h at 28°C, and the number of cfu were thereafter determined by counting visible colonies. Activity of 30 *μ*M HKH20, GKH17, GKH17WWW, and GKH17WWWWW peptides against *Candida* was also tested in physiological salt conditions (0.15 M NaCl in 10 mM Tris, pH 7.4) in presence of 20% as well as 50% human plasma, and 50% human serum (in 0.15 M NaCl, 10 mM Tris, pH 7.4) (Figure 3). The controls (Figure 3) defined the total (100%) survival of *Candida* cells incubated in the same buffer under identical conditions but without peptides. To test the time dependence of fungal killing, *Candida* was subjected to 30 *μ*M of HKH20 peptide and incubated for 5, 10, 15, 30, 60, and 150 minutes (Figure 2(d)).

Radial diffusion assay (RDA) was performed as described by Lehrer et al. [39] with some minor modifications. *Candida *was grown for 16 h at 28°C in 10 mL YPD to obtain yeast form organisms. The culture was centrifuged at 2000 ×g for 10 min then washed three times with 10 mM Tris, pH 7.4. A volume containing 1 × 10^5^ cfu was added to 5 mL of previously autoclaved, 42°C, underlay agarose gel that contained 1.5 mg of Tryptic Soy Broth (TSB) base, 50 mg of low-electroendosmosistype (Low-EEO) agarose type I (A-6013-25G) (Sigma-Aldrich Inc, St. Louis, Mo, USA), and 1 *μ*L of Tween 20 (0.02% w/v) (Sigma Chemical Co.) dissolved in 10 mM Tris, pH 7.4. The fungi were directly dispensed into the agar and mixed with a vortex mixer for 10 seconds and then quickly poured out into a petri dish, 85 mm in diameter. Wells, 4 mm in diameter, were punched, and each well was then filled up with 6 *μ*L of test sample. After incubation at 35–37°C for 3 h, 5 mL of overlay agarose gel, consisting of 300 mg TSB, 50 mg Low-EEO agarose dissolved in distilled sterilized water, was poured out on top of the underlay gel. The clear zone surrounding the wells, indicating antifungal activity, was measured after 18–24 h of incubation at 28°C. HMWK degradation products were tested for RDA inhibitory effects (Figure 1(b)). Human leukocyte (neutrophil) elastase (HLE) and freeze-thaw-disrupted polymorphonuclear neutrophils (PMNs) at equivalent concentrations as used in the HMWK degradation were incubated at 37°C for 60 minutes and added as controls (Figure 1(b)). 

*Malassezia furfur* ATCC 44342 (CCUG 24230) was obtained from the Department of Bacteriology at Skåne University Hospital. The antifungal activity was tested in an assay described by Lopez-Gracia et al. [40], but with some minor modifications. Cells were cultured for 2 days at 37*º*C in 10 mL medium containing 4% Bacto Malt Extract (Becton, Dickinson and Company, Md, USA), 1% glucose (VWR international), 1% Tween 80 (Sigma-Aldrich, St. Louis, Mo, USA) and 0.6% Bacto Peptone (Becton, Dickinson and Company, Md, USA). Cells were then washed in 1 mM sodium phosphate buffer, pH 7.4. *M. furfur* cells were grown in sterile 96-well microtiter plates (Costar, Corning Incorporated, Corning, USA) in a total volume of 100 *μ*L, containing 60 *μ*L of 1 mM sodium phosphate buffer, pH 7.4, 20 *μ*L of culture medium, 10 *μ*L of a 10x stock solution of the synthetic peptides, and a 10x stock solution of the fungal cell suspension, resulting in a final concentration of 1-2 × 10^5^ cells/mL. Chloramphenicol was added to each well (16 *μ*g/ml) to avoid contamination. The cells were incubated at 30°C for 24 h. To quantify the fungicidal activity, serial dilutions of the incubation mixture were plated onto agar plates and incubated 72 h at 37°C, and the number of cfu thereafter was determined by counting visible colonies. The minimal inhibitory concentration (MIC) was defined as the concentration which yielded at least 95% inhibition of fungal growth.

### 2.4. Fluorescence Microscopy

*C. albicans *ATCC 90028 was incubated for 18 hours in 10 mL YPD-broth at 28°C to obtain midlogarithmic phase organisms and washed three times, then diluted in 10 mM Tris, pH 7.4, to a concentration of 1 × 10^7^ cfu/mL. Two hundred *μ*L of the *C. albicans *suspension was incubated for 5 min on ice together with 1 *μ*L Texas Red-conjugated HKH20 (2 mg/mL). Not only to block binding of HKH20 to the fungal membrane, but also to exclude the possibility that the Texas Red-label confers unspecific binding to fungal surfaces, 2 *μ*L heparin (50 mg/mL) was added to the samples prior to incubation with Texas Red-conjugated HKH20. The pellets were washed three times in 10 mM Tris, pH 7.4. Cell FIX (Becton Dickinson Catalogue no. 340181) was added (to a final concentration of 4% formaldehyde), and the yeast cell pellets were incubated 15 min on ice and then 45 min at room temperature. Ethanol washed glass cover slips were coated with 0.25 mL of poly-lysine (0.2 mg/mL dissolved in water), dried, and then washed with distilled water. The samples were adhered to the polylysine glasses for 30 min and mounted on microscope slides for visual inspection. A Nikon Eclipse TE300 inverted fluorescence microscope was used.

### 2.5. Liposome Preparation and Leakage Assay

The liposomes investigated were zwitterionic DOPC/cholesterol (60/40 mol/mol) or DOPC/ergosterol (60/40 mol/mol). DOPC (1,2-Dioleoyl-sn-Glycero-3-Phosphatidylcholine) was from Avanti Polar Lipids (Alabaster, USA) and of >99% purity, while ergosterol and cholesterol (>99% purity) were from Sigma-Aldrich (St. Louis, USA). Due to the long, symmetric, and unsaturated acyl chains of DOPC, several methodological advantages are reached. In particular, membrane cohesion is good, which facilitates very stable unilamellar liposomes, allowing detailed values on leakage to be obtained. The lipid mixtures were dissolved in chloroform, after which solvent was removed by evaporation under vacuum overnight. Subsequently, 10 mM Tris buffer, pH 7.4, was added together with 0.1 M carboxyfluorescein (CF) (Sigma, St. Louis, USA). After hydration, the lipid mixture was subjected to eight freeze-thaw cycles consisting of freezing in liquid nitrogen and heated to 60°C. Unilamellar liposomes of about Ø140 nm were generated by multiple extrusions through polycarbonate filters (pore size 100 nm) mounted in a LipoFast miniextruder (Avestin, Ottawa, Canada) at 22°C. Untrapped CF was removed by two subsequent gel filtrations (Sephadex G-50, GE Healthcare, Uppsala, Sweden) at 22°C, with Tris buffer as eluent. CF release from the liposomes was determined by monitoring the emitted fluorescence at 520 nm from a liposome dispersion (10 *μ*M lipid in 10 mM Tris, 5 mM glucose, pH 7.4). An absolute leakage scale was obtained by disrupting the liposomes at the end of each experiment through addition of 0.8 mM Triton X-100 (Sigma-Aldrich, St. Louis, USA). A SPEX-fluorolog 1650 0.22-m double spectrometer (SPEX Industries, Edison, USA) was used for the liposome leakage assay. Measurements were performed in at least duplicate at 37°C.

### 2.6. Statistical Analysis

Comparison of antifungal activity between the various peptides was performed using Kruskal-Wallis One-Way Analysis of Variance on Ranks and pair-ways multiple-comparison procedures (Tukey Test). *P* < 0.05 was considered as a significant difference. The statistical software used was SigmaStat (Systat Software Inc., Point Richmond, Calif, USA).

## 3. Results

### 3.1. Proteolytic Cleavage of HMWK Generates Peptides with Antifungal Properties

In order to test whether antifungal peptides could be released after proteolytic digestion of HMWK *in vivo*, HMWK was incubated with neutrophil elastase or extracts of polymorphonuclear neutrophils. Analysis using SDS-PAGE revealed that HMWK was degraded into several fragments (Figure 1(a)). Furthermore, when the fragments were analysed in RDA, antifungal activity was observed (Figure 1(b)). In analogy to the above, HMWK incubated with Cathepsin G resulted in the generation of low molecular-weight fragments corresponding to the previously detected 20-mer peptide from D5, HKH20, as assessed by Western blot using polyclonal antibodies against HKH20 (Figure 1(c)).

### 3.2. Antifungal Effects of rD5 and the D5-Derived Peptide HKH20 from HMWK

To investigate whether domain 5 has antifungal activity, effects of purified rD5 on *C. parapsilosis* ATCC 90018 were tested in a fungicidal assay (concentrations ranging from 0.3 *μ*M to 60 *μ*M) and in RDA. The results showed a dose-dependent killing of *Candida *(Figure 2(a)), and, notably, rD5 exerted inhibitory activity on *Candida* in RDA similar to the reference peptides LL-37 and histatin-5 (Figure 2(b)). In order to determine possible antifungal peptides of the rD5, overlapping peptides spanning the D5-region were synthesized (Table 1). When HKH20 and GKH17 were tested in RDA, a significantly more potent inhibitory activity on *Candida*, in comparison to LL-37, was observed (Figure 2(c)). As shown, HKH20 possessed potent antifungal activity (Figure 2(a)), and >80% of the *Candida* cells were killed by 30 *μ*M HKH20 within 15 minutes (Figure 2(d)). In RDA, HKH20 displayed significantly higher inhibitory activity on *Candida*, when compared with LL-37 (Figures 2(b) and 2(c)). Finally, *M. furfur* was tested in an antifungal assay. The yeast cells were subjected to 10 *μ*M or 30 *μ*M of LL-37, HKH20, or GKH17, and the results showed that particularly HKH20 and GKH17 were antifungal at 10 and 30 *μ*M (Figure 2(e)). MIC values, defined as the concentration yielding at least 95% inhibition of fungal growth, for HKH20 and GKH17 were determined to be between 2.5–5 *μ*M and 5–10 *μ*M, respectively (Figure 2(f)).

### 3.3. The D5-Derived Peptide HKH20 Binds to the Surface of Candida

To elucidate whether the peptide HKH20 interacts with the fungal cell wall of *Candida*, HKH20, labeled with the fluorescent dye Texas Red, was incubated with the fungal cells. As demonstrated by fluorescence microscopy, the peptide bound to the surface of *Candida* and the binding were completely blocked by heparin, an anionic glycosaminoglycan previously shown to block the antibacterial activity of HKH20 [33] (Figure 2(g)).

### 3.4. Antifungal Effects of D5-Derived Peptides and Structural Modifications for an Optimized Effect at Physiological Conditions

Hydrophobic tagging with W (Trp) amino acid residues has previously been demonstrated to increase antibacterial activity against various pathogens, such as *S. aureus* and *P. aeruginosa*. Particularly, for short, highly positively charged, and hydrophilic peptides, this facilitates the design of potent AMPs that selectively bind microorganisms over human cells [41–43]. Thus, the GKH17 peptide was tagged with 3–5 W residues at the C-terminal and analysed for antifungal activity. As shown, the hydrophobic modification resulted in increased antifungal activity, which was observed when not only in low salt conditions, but also tested in physiological salt solution (0.15 M NaCl) as well as in the presence of human plasma and serum (20% and 50%) (Figure 3).

### 3.5. Membrane-Disruptive Potency of GKH17 and End-Tagged Variants

**Figure 4:**
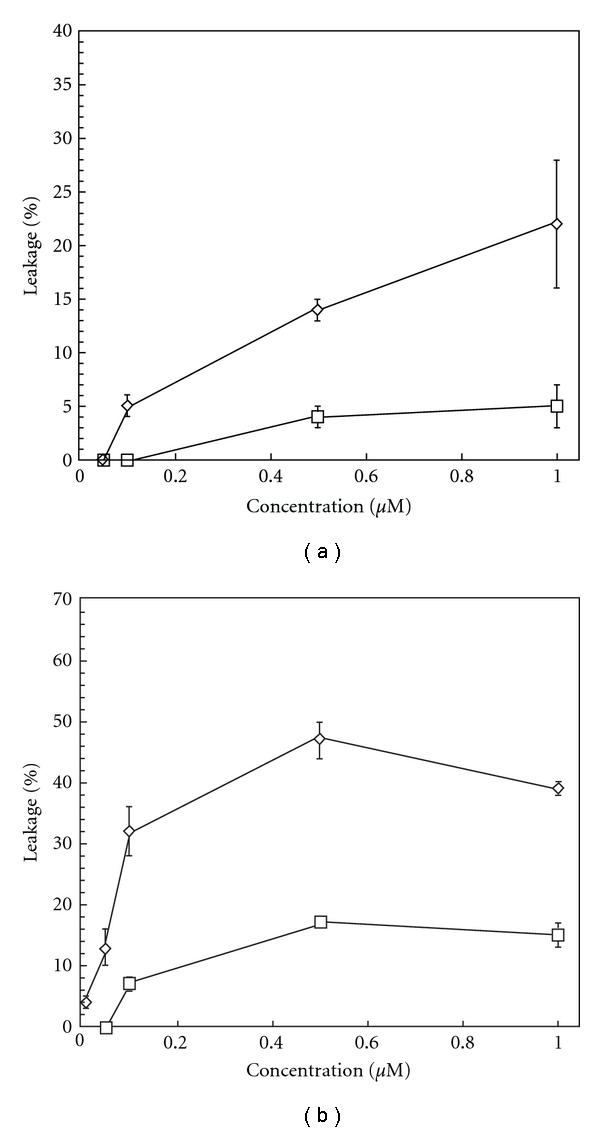
Effects of GKH17 (a) and GKH17WWW (b) on liposome permeability. For both peptides (at the doses indicated), permeabilization of ergosterol-containing liposomes (open circle) is demonstrated to be higher than that of cholesterol-containing (open square) ones.

The membrane-disruptive potency for GKH17 and the end-tagged variant GKH17WWW were demonstrated to be higher for ergosterol-containing liposomes than for cholesterol-containing ones (Figure 4). This is in line with the membrane-condensing effect of cholesterol [44–48], which opposes membrane insertion of peptides, and the relatively smaller membrane condensation caused by ergosterol. Given this difference between cholesterol and ergosterol, identification of peptides which are selective for ergosterol-containing membranes may represent a means to achieve simultaneously antifungal and low-toxic therapeutics.

## 4. Discussion

The D5 domain of HMWK is multifunctional and may participate in cell binding [49], inhibit angiogenesis, proliferation, and migration of endothelial cells [50, 51], and act as an antibacterial agent [33]. The main result in the present study is that cleavage products of HMWK also act as antifungal peptides, paralleled by results showing that the D5 domain of HMWK and related amino-acid sequences exert an antifungal activity comparable with the well-known AMPs LL-37 and histatin 5. Innate immune responses, including small cationic peptides with antimicrobial activities, constitute the first line of defense and are crucial for rapid clearing of microbial invaders. Numerous antimicrobial and antifungal peptides have been identified to date, most of them acting by membrane disruptive mechanisms. As mentioned briefly in the Introduction, biological effects exerted by AMPs include growth stimulus and angiogenesis, protease inhibition, antiangiogenesis, and chemotaxis [52–54]. Conversely, cationic peptide motifs from proteins not previously considered as AMPs have been shown to exert antimicrobial activities. For example, complement C3 [55, 56], kininogen [34, 57], heparin-binding protein [58], heparin-binding epidermal growth factor and other growth factors [59], matrix proteins such as laminin, fibronectin and proline arginine-rich and leucine-rich repeat protein (PRELP) [60], prions [61], *β*2-glycoprotein [62], histidine-rich glycoprotein [10], thrombin [63], and tissue factor pathway inhibitor [64] may act either as holoproteins or smaller peptide derivatives or fragments thereof and also exert antimicrobial activities *in vitro*, and, in several cases, *in vivo * [10, 34, 57]. In general, these findings are compatible with the observation that consensus heparin-binding peptide sequences (Cardin and Weintraub motifs) XBBBXXBX or XBBXBX (where X represents hydrophobic or uncharged amino acids, and B represents basic amino acids), represented by multiples of the motifs ARKKAAKA or AKKARA [65], are antibacterial [20] and specifically interact with membranes [66].

The findings in this study therefore further underline the multifunctionality of AMPs and illustrate that the glycine- and histidine-rich part of D5, previously shown to interact with heparin, may bind to negatively charged fungal membranes, such as those of *Candida* and *Malassezia* [67–70]. It is also notable that HKH20, derived from the lysine- and histidine-rich region of D5, which comprises consensus sequences for heparin binding [20, 65], binds to the membrane of* Candida*, likely by interacting with negatively charged molecules at the fungal cell wall [71]. Interestingly, HKH20 resembles the H-and K-rich AMP histatin-5 (Table 1), an AMP known to mediate fungal killing. It is also worth noting that the light chain of HMWK is essential for the interaction of kininogen with the cell wall of *Candida* [32]*.*

 As previously demonstrated, end-tagging by hydrophobic amino acid stretches is a facile and flexible approach of general applicability, by which AMP boosting, particularly of linear cationic sequences, is obtained [72, 73]. The data demonstrating that the hydrophobically modified GKH17 peptides show enhanced activity at physiological conditions, and in presence of human plasma and serum are therefore well in line with these previous data, suggesting that increased hydrophobicity yields increased affinity for fungal membranes. Indeed, these data parallel the observations on liposomes, demonstrating specificity for ergosterol (fungi-like) liposomes compared with cholesterol (mammalian cell mimicking) liposomes. 

 In previous reports, it was demonstrated that fragments of HMWK are generated upon degradation with neutrophil elastase as well as bacterial enzymes [74]. The observation in the present study extend these previous findings and demonstrate that cathepsin G, a major neutrophilic enzyme, may also degrade HMWK, leading to release of AMPs (Figure 1(c)). Considering these data as well as the finding that PMNs may degrade HMWK and generate antifungal activity (Figure 1(b)), it is tempting to speculate that during a cutaneous *Candida* infection, neutrophils, recruited to the site of infection [75, 76], may upon activation release cathepsin G from azurophilic granules, leading to degradation of HMWK, present in blood or connective tissues. The cleavage products of HMWK can then act as antifungal AMPs and inhibit fungal cells at the site of infection. However, further investigations are clearly mandatory in order to further define the generation of D5 peptides in response to fungal infection.

 In conclusion, we have shown that proteolysis of HMWK generates fragments with antifungal activities. Amino-acid sequences corresponding to D5-derived peptides exert antifungal effects, and, furthermore, the antifungal activity may be enhanced by tagging with hydrophobic amino-acid residues.

## Figures and Tables

**Table 1:** D5-derived and control peptides investigated.

Peptide	Sequence	Mw	pI^1^	Net charge	Mean hydrophobicity^2^
KHN20	KHNLGHGHKHERDQGHGHQR	2365.5	9.99	+2	−4.91
GHG20	GHGLGHGHEQQHGLGHGHKF	2127.2	7.21	0	−2.01
GHG21	GHGHKFKLDDDLEHQGGHVLD	2354.5	5.63	−3	−2.32
GGH20	GGHVLDHGHKHKHGHGHGKH	2169.3	9.70	+2	−3.44
HKH20	HKHGHGHGKHKNKGKKNGKH	2151.5	10.78	+7	−5.91
GKH17	GKHKNKGKKNGKHNGWK	1946	10.78	+7	−5.77
GKH17WWW	GKHKNKGKKNGKHNGWKWWW	2505	10.78	+7	−3.45
GKH17WWWWW	GKHKNKGKKNGKHNGWKWWWWW	2287	10.78	+7	−2.25
LL-37	LLGDFFRKSKEKIGKEFKRIVQRIKDFLRNLVPRTES	4492	10.61	+6	−1.84
Histatin-5	DSHAKRHHGYKRKFHEKHHSHRGY	3036	10.28	+5	−4.67

Mw: molecular weight. ^1^pI: theoretical isoelectric point calculated by using the Protparam tool available at http://us.expasy.org/tools/protparam.html.^ 2^Mean hydrophobicity calculated using HydroMCalc (combined consensus hydrophobicity scale) available at http://www.bbcm.univ.trieste.it/~tossi/HydroCalc/HydroMCalc.html.
